# A generic algorithm for layout of biological networks

**DOI:** 10.1186/1471-2105-10-375

**Published:** 2009-11-12

**Authors:** Falk Schreiber, Tim Dwyer, Kim Marriott, Michael Wybrow

**Affiliations:** 1 Leibniz-Institute of Plant Genetics and Crop Plant Research (IPK), Corrensstrasse 3, D-06466 Gatersleben, Germany; 2 Institute of Computer Science, Martin Luther University Halle-Wittenberg, Von-Seckendorff-Platz 1, D-06120 Halle, Germany; 3 Microsoft Research, Seattle, USA; 4 Clayton School of Information Technology, Monash University, Vic 3800, Australia

## Abstract

**Background:**

Biological networks are widely used to represent processes in biological systems and to capture interactions and dependencies between biological entities. Their size and complexity is steadily increasing due to the ongoing growth of knowledge in the life sciences. To aid understanding of biological networks several algorithms for laying out and graphically representing networks and network analysis results have been developed. However, current algorithms are specialized to particular layout styles and therefore different algorithms are required for each kind of network and/or style of layout. This increases implementation effort and means that new algorithms must be developed for new layout styles. Furthermore, additional effort is necessary to compose different layout conventions in the same diagram. Also the user cannot usually customize the placement of nodes to tailor the layout to their particular need or task and there is little support for interactive network exploration.

**Results:**

We present a novel algorithm to visualize different biological networks and network analysis results in meaningful ways depending on network types and analysis outcome. Our method is based on constrained graph layout and we demonstrate how it can handle the drawing conventions used in biological networks.

**Conclusion:**

The presented algorithm offers the ability to produce many of the fundamental popular drawing styles while allowing the exibility of constraints to further tailor these layouts.

## Background

Networks play a central role in biological investigation of organisms. They are used to represent processes in biological systems and to capture interactions and dependencies between biological entities such as genes, transcripts, proteins and metabolites. One large application area for network-centered analysis and visualization is Systems Biology, an increasingly important research field which aims at a comprehensive understanding and remodeling of the processes in living beings [1,2]. Due to the steady growth of knowledge in the life sciences such networks are increasingly large and complex. To tackle this complexity and help in analyzing and interpreting the complicated web of interactions meaningful visualizations of biological networks are crucial.

Methods for automatic network visualization have gained increased attention from the research community over recent years and various layout algorithms have been developed, e. g. [3-11]. Often standard layout methods such as force directed [12,13], layered [14,15] and circular [16] approaches are used to draw these networks. However, the direct use of standard layout methods is somewhat unsatisfactory since biological networks often have specialized layout requirements reflecting the drawing conventions historically used in manually laid out diagrams (which have been developed to better emphasize relevant biological relationships and concepts). This has led to the development of network- and application-specific layout algorithms, for example, for signal transduction maps [17,18], protein interaction networks [3,6], metabolic pathways [4,10,19] and protein-domain interaction networks [20]. Advanced solutions combine different layout styles (such as linear, circular and branching layouts) for sub-networks or use specific layouts styles for particular network parts such as cycles [7,10,21].

However, current approaches for the automatic visualization of biological networks have four major drawbacks resulting from the specialized nature of these algorithms:

1. Different kinds of biological networks (e. g. protein interaction or metabolic networks) have different layout conventions and this requires the implementation and sometimes development of specialized layout algorithms for each convention.

2. It is not easy to combine networks with different layout conventions in the one drawing since the layout algorithms use quite different approaches and so cannot be easily combined.

3. The user cannot tailor the standard layout algorithms for their particular need or task by e. g. emphasizing the pathways of interest by making them straight.

4. The algorithms do not sufficiently support interactive network exploration. Usually with these algorithms small modifications in the network structure and re-layout of the network results in very different pictures. However, such sudden and large changes destroy the user's mental map (i. e. the user's understanding of the network based on the previous view) and therefore hinder interactive understanding of the network.

Here we present a new algorithm for layout of biological networks that overcomes these limitations. It is based on a powerful new graph drawing technique, *constrained graph layout *[22]. Like force-directed layout [12,13] constrained graph layout works by minimizing an objective function that measures the quality of the layout. However it extends force-directed layout by allowing minimization of the objective to be done subject to placement constraints on the objects in the network. This is achieved by using mathematically rigorous optimization techniques based on gradient projection [23]. Efficient implementation is made possible by restricting the placement constraints to be *separation constraints *of the form *u *+ *g *≤ (=) *v*, enforcing a minimum (or precise) gap *g *between the positions *u *and *v *of pairs of objects in either the *x *or *y *dimensions of the drawing.

A significant contribution of this paper is to show that separation constraints, despite their apparent simplicity and their limitation to act on a single dimension can in fact be used to encode the wide variety of specialized layout requirements arising in biological networks. Examples of such requirements are placement of nodes below other nodes in directed graphs, drawing cycles on a rectangle, alignment of nodes, non-overlap of nodes, orthogonal ordering between nodes, containment of nodes in clusters, standard layout of motifs and containment in a page. A key technique is to generate separation constraints that approximate complex non-linear constraints such as non-overlap and to update this approximation dynamically as the final layout is computed. Furthermore, these separation constraints can be automatically derived from the visualization requirements, network analysis results and interactive network changes. With this algorithm it is possible to obtain layout results which are close to the results of different existing layout algorithms.

The presented approach provides a generic, universal algorithm for layout of biological networks:

1. It greatly simplifies the implementation of layout methods for life sciences, systems and synthetic biology tools, which have previously had to utilize very different layout algorithms for different types of biological networks (or different layout requirements).

2. It allows the use of different layout styles for different parts of one large network.

3. It allows the user to customize the layout by adding separation constraints.

4. It lends itself to mental-map-preserving dynamic layout in interactive systems, thereby supporting interactive exploration of large and complex networks.

This paper is structured as follows: in the Methods section we introduce some terminology, detail the constrained graph layout method, present the kinds of placement constraints that are needed to fulfill layout requirements of different biological networks and discuss how they can be automatically generated and then implemented in terms of the separation constraints supported by the layout method. The Results section provides examples of the layout method for a number of different kinds of networks and shows its advantages over current layout methods. Finally, the Conclusion contains the discussion and some suggestions for future work.

## Methods

### Layout Framework

#### The layout problem

A *network *(or *graph*) *G *= (*V*, *E*) contains a finite set of nodes *V *and a finite set of edges *E *⊆ {(*u*, *v*) | *u*, *v *∈ *V*}. Biological networks can contain undirected and/or directed interactions. Therefore we consider undirected, directed and mixed graphs, and an edge may be undirected or directed. A *layout L *of a network *G *assigns coordinates to the nodes and a path to each edge. A *partial layout P *= (*x, y*) of a network *G *consists of just an assignment of node positions, where (*x*_*u*_, *y*_*u*_) is the position of node *u*. While we focus on 2D layout our method can be easily extended to 3D layout.

The basic approach in constrained graph layout (like force-directed layout) for finding aesthetically pleasing drawings of graphs is to define a *cost function F*(*P*) over the positions of the nodes *P *and then to minimize this cost function by adjusting the positions. One commonly used cost function in force-directed layout is the *stress *function [24]:(1)

where . This tries to place each pair of nodes *u*, *v *their ideal distance apart *d*_*uv *_which is proportional to the length of the shortest path between the nodes). It measures the sum of squared differences between the ideal spacing for each pair of nodes and their Euclidean distance in the layout. While this is the cost function we have used, we wish to emphasize that our technique is generic in the choice of cost function.

The main difference between force-directed layout and constrained graph layout is that in constrained graph layout the layout algorithm is required to satisfy *placement constraints *on the nodes, such as non-overlap of the nodes or node alignment. We say that a layout is *feasible *if it satisfies the placement constraints.

The layout problem is to find a layout *L *for a graph *G *that is feasible and which is locally optimal in the sense that moving the nodes slightly either leads to infeasibility or increases the cost function.

#### Layout method

**Figure 1:**
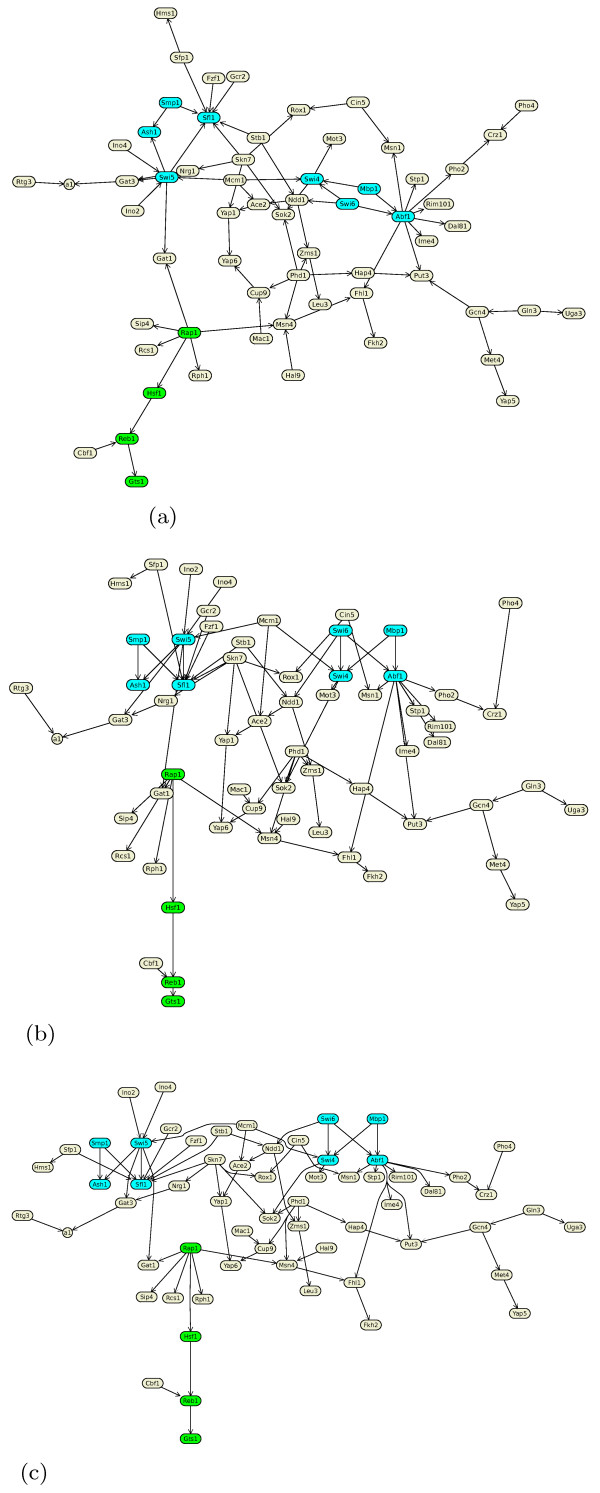
**Layout steps: Our layout method involves taking an (a) initial layout, (b) Finding a feasible layout that satisfies all the placement constraints, and performing gradient projection to produce (c) a final optimized layout**. This gene regulatory network has two bi-fan motifs drawn similarly and one path emphasized via constraints.

The basic layout method has three main steps (as shown in Figure 1):

1. Find a feasible partial layout *P*_*feas *_satisfying all placement constraints.

2. Starting from *P*_*feas *_perform gradient projection to find a locally optimal partial layout *P*_*opt*_.

3. Extend *P*_*opt *_to a full layout *L *by computing paths for edges.

**Figure 2:**
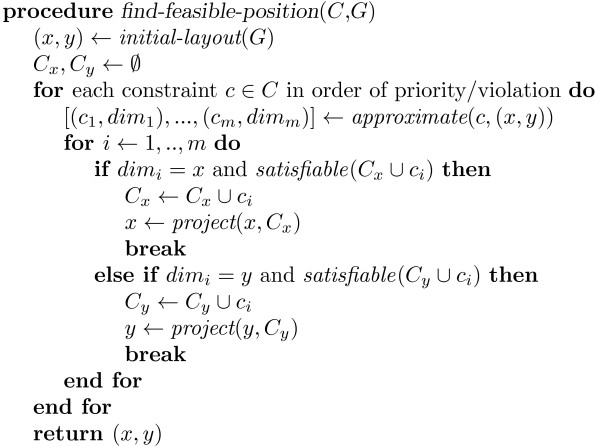
**Procedure *find-feasible-position *(*C *- set of constraints, *G *- graph); see text for details**.

Step 1 (*find-feasible-position*, see Figure 2) starts with an initial position (*x*, *y*) for the nodes found by a force-directed layout method since this gives a reasonable "default" position for the nodes that reflects the basic graph structure. This position is then iteratively updated with a greedy heuristic, so as to satisfy more and more of the placement constraints.

Here *G *is the graph, *C *is the set of constraints and *C*_*x *_and *C*_*y *_are sets of separation constraints (for the *x *and *y *dimension, resp.) that enforce the placement constraints enforced so far. The function *approximate*(*c*, (*x*, *y*)) returns pairs of sets of separation constraints *c*_*i *_and their respective dimension *dim*_*i *_that will enforce satisfaction of the violated constraint *c*. Each *c*_*i *_implies the placement constraint and the first *c*_*i *_that can be added while maintaining feasibility is chosen. In the case that all *c*_*i *_lead to infeasibility none is added and the constraint *c *is effectively ignored.

The function *project *returns positions for nodes which satisfy the separation constraints and which are as close as possible to the current position. Projection is performed on a single dimension at a time. This is possible since the separation constraints for each dimension are independent. More precisely, *project*(*d*, *C*) returns *x *= min_*x*_Σ_*v*∈*V *_(*x*_*v *_- *d*_*v*_)^2 ^*subject to C*. This is done using the algorithm from [25].

**Figure 3:**
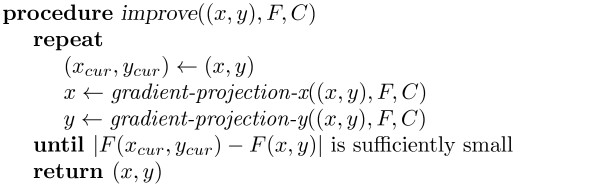
**Procedure *improve *((*x*, *y*) - initial position for the nodes, *F *- cost function, *C *- set of constraints); see text for details**.

Step 2 (*improve*, see Figure 3) takes an initial position *P *= (*x*, *y*) for the nodes, a set of placement constraints *C *and a cost function *F*. It works by alternately adjusting horizontal and vertical positions of all nodes to incrementally reduce the cost function. Again this is possible because the separation constraints in each dimension are independent. This makes the computation of the new positions considerably simpler than if both dimensions had to be considered together. The high-level algorithm is given in Figure 3.

The adjustment step in each dimension is performed by *gradient-projection-x *and *gradient-projection-y*. We only consider *gradient-projection-x *since the two routines are entirely symmetric. The routine *gradient-projection-x *decreases the cost function *F *by moving nodes horizontally in the direction of steepest descent from the current node position (*x*_*cur*_, *y*_*cur*_). Computation of the step size depends on the cost function. However we have had success by simply using a quadratic approximation based on the second order Taylor series expansion of *F *around *x *and choosing the value of the step size that minimizes this approximation.

Of course the desired horizontal position for the nodes is not guaranteed to satisfy the placement constraints. To remedy this the algorithm generates a set of horizontal separation constraints *C*_*x *_from the placement constraints *C *that safely approximate the placement constraints in the sense that *C*_*x*_(*x*) ⇒ *C*(*x*, *y*_*cur*_) and *x*_*cur *_satisfies *C*_*x*_. The new horizontal position *x *is obtained by calling *project *to projecting the desired position *d *on to *C*_*x*_.

In Step 3 edges can be drawn using straight-lines or any other desired style such as poly-line routings [26,27]. We note that the edge-routing library libavoid which implements the method described in [27] has been extended to handle clusters and finds routes for edges that do not unnecessarily pass through clusters. It can also perform "nudging" on the final routes to separate paths with shared sub-routes.

### Placement Constraints

In this section we show that our approach of dynamically generating separation constraints is very powerful and supports the kinds of placement constraints arising in biological networks. We then discuss which placement constraints are used for different layouts and how these constraints can be derived from biological (network) information.

**Figure 4:**
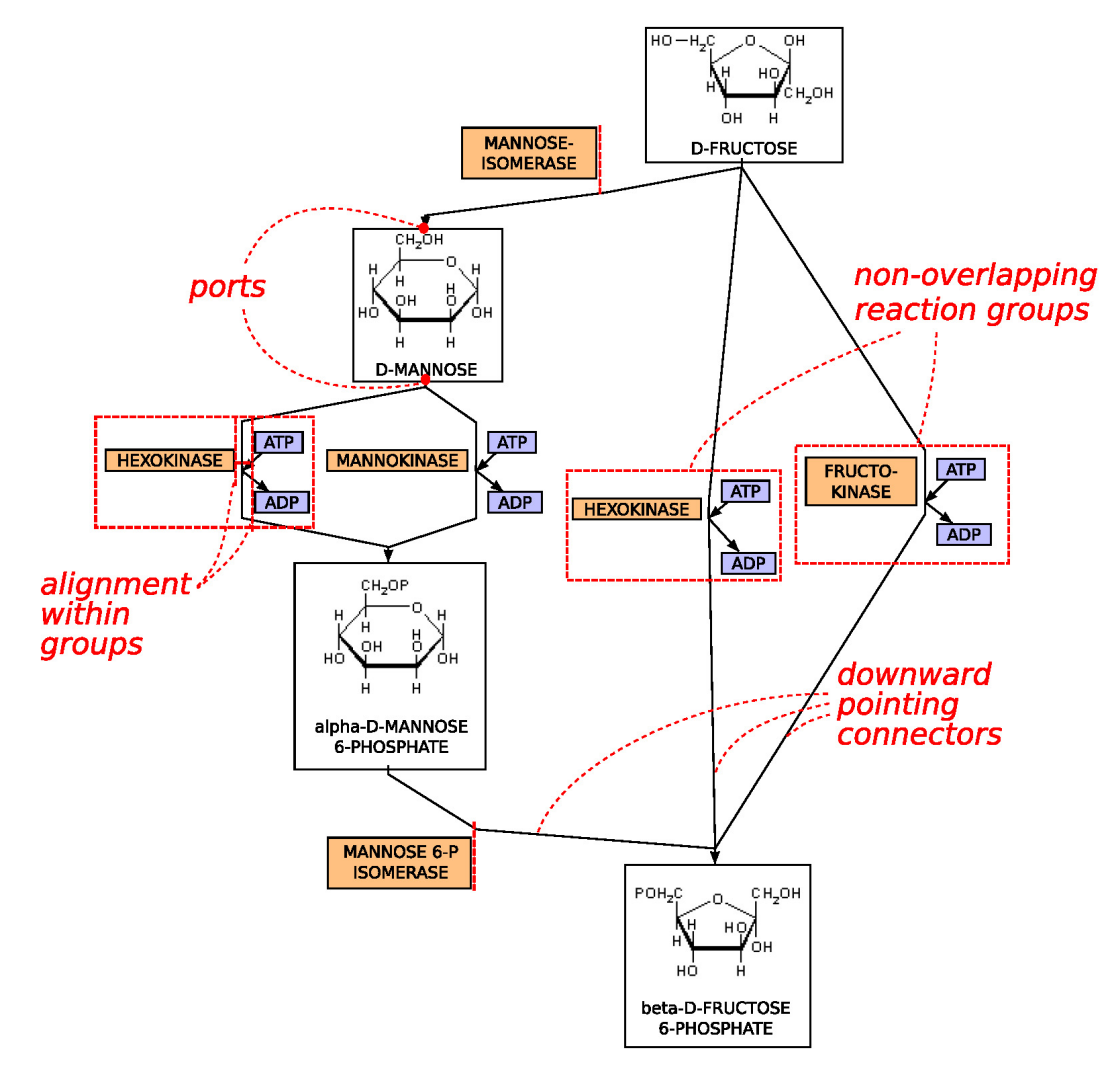
**A metabolic pathway arranged with standard drawing conventions emphasized using various constraints**. Metabolic pathways show chemical reactions occurring within a cell.

Figure 4 gives a general idea of how constraints can be used to arrange network elements. For example, parts of reactions such as enzymes and co-reactants should be close together and are clustered into non-overlapping reaction groups, where all nodes are aligned within the group. The nodes are arranged such that the reactions flow in a particular direction as much as possible. Note that these high-level constraints are internally represented by sets of separation constraints.

The following placement constraints are major examples of high-level constraints which can be solved by our algorithm.

#### Pathway emphasis

Often some paths within a network are of special interest. These can be emphasized by using separation constraints to horizontally or vertically align the nodes in the pathway (as shown in Figure 1). For example, in a metabolic network the path with the highest flux may be determined by computational (e. g. Flux Balance Analysis [28]) or experimental methods and then automatically highlighted in this manner.

#### Directed edges

**Figure 5:**
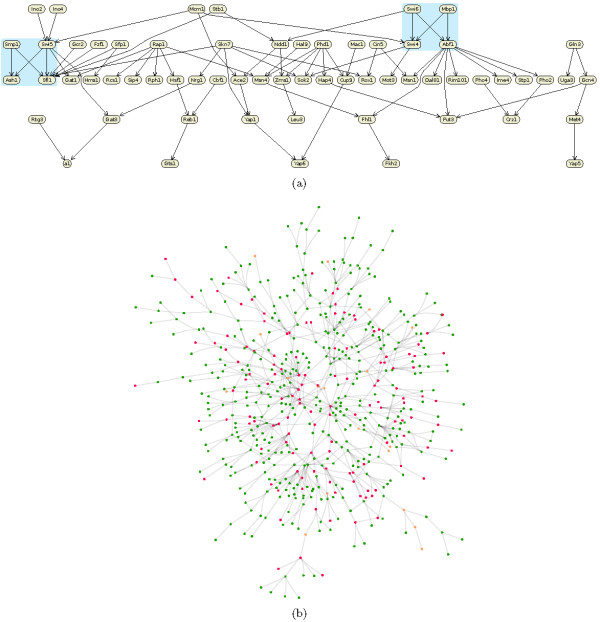
**The presented method provides a generic approach to network visualization**. It produces the drawings above which previously had to be produced using two totally different algorithms: (a) is a drawing of a gene regulatory network (showing the indirect interaction of genes through their RNA and protein expression products) using a Sugiyama layered layout style (note the two bi-fan motifs highlighted and drawn similarly), (b) is a protein interaction network (showing the interaction of proteins in a cell) using a force-directed layout style (Figure 7 shows the affect of adding constraints to this layout).

Often networks contain directed edges, and edge direction is used to encode flow of information or material. Direction of edges can be emphasized by constraints requiring that the start node of the edge is above (or to the left of) its end node. See Figures 4 and 5(a) for examples. The information about edge direction can be derived from a directed network by first removing cycles in the network using a decycling algorithm [29] and than using the direction of the edges in the acyclic directed network as constraints.

#### Cycles

**Figure 6:**
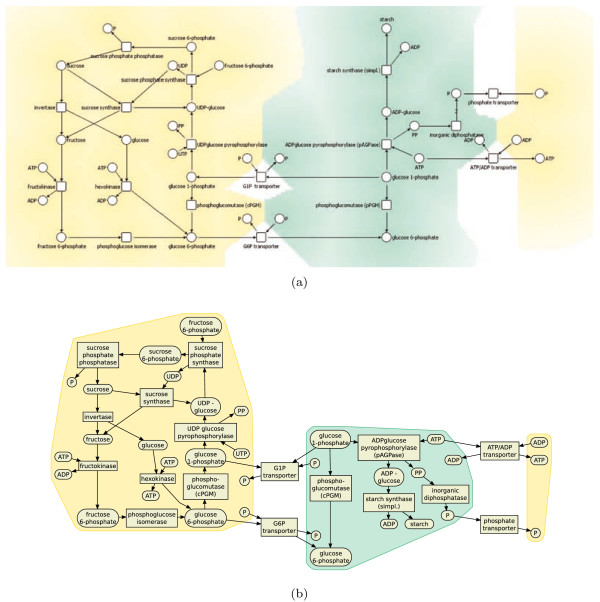
**Handling of convex and rectangular clusters allows network hierarchy to be emphasized**. (a) shows an example a metabolic pathway (showing chemical reactions occurring within a cell, in this example a part of the Glycolysis and Gluconeogenesis pathway is used) with compartments manually drawn (manual layout derived from the MetaCrop database [30]), (b) shows the same network drawn automatically.

Cycles occur, for example, in metabolic networks (e. g. TCA cycle, urea cycle) and are usually specially arranged to emphasize the cyclic processes. Cycles can be emphasized in a number of different ways. The first way is to introduce a dummy node in the center of the cycle and attach a strongly weighted dummy edge from the center node to each node in the cycle. This will have the effect of arranging the nodes in the cycle around a circle. The second way is to arrange the nodes on the perimeter of a rectangle (as in Figure 6(b)). To do so we introduce new variables *x*_*l*_, *x*_*r*_, *y*_*b*_, *y*_*t *_corresponding to the four sides of the rectangle. The placement constraint that node *v *lies on the rectangle is approximated by one of four conjunctions of separation constraints one for each side of the rectangle. For instance, *y*_*v *_= *y*_*b *_∧ *x*_*l *_≤ *x*_*v *_∧ *x*_*v *_≤ *x*_*r *_constrains node *v *to be on the bottom of such a rectangle. Such constraints can be derived by cycle detection algorithms such as [21] or by additional information about cycles in pathway databases (e. g. in MetaCrop [30] and BioPath [31]).

#### Emphasizing network motifs

Different occurrences of a network motif should be drawn in the same way. Equality separation constraints can be used to force all occurrences of a particular motif to be drawn in exactly the same way, see Figure 5(a) for an example. Different occurrences of a network motif can be computed with motif-detection algorithms such as [32,33], the equality constraints are than derived from a given or pre-computed layout of this motif and transferred to all occurrences of the motif.

#### Clusters and compartments

Often biological networks contain node clusters. Containment within a rectangular region is simple to model using separation constraints. By introducing a rectangle for compartments or clusters in the graph we can group nodes together. It is then natural to modify the cost function so that it tries to reduce the width and height of this rectangle to size proportional to the number of nodes in the compartment. The cluster boundary is obtained by either taking the convex hull of the nodes in the cluster or the bounding rectangle. An example is shown in Figure 6. Such clustering (and therefore the cluster constraint) may be specified by biological information such as cellular compartments or be computed by clustering algorithms [34].

#### Non-overlap of nodes and compartments

A common problem with general purpose layout engines is that nodes are treated as points and so, if nodes are large as is the case in many biological networks, they may overlap. Non-overlap of nodes is readily handled in our approach by approximating the placement constraint that nodes *u *and *v *do not overlap by the disjunction of four separation constraints: *u *left of *v*, *u *above *v*, *v *left of *u *or *v *above *u*. Similarly non-overlap of compartments can be handled. For efficiency generation of separation constraints to enforce non-overlap in single dimension is done using the scan-line algorithm given in [35] which generates a linear number of constraints.

#### Orthogonal ordering of nodes and layout stability

Preserving the relative horizontal and vertical ordering of nodes as a layout changes can help preserve the users mental map of the layout. Layout stability is also aided by adding terms to the cost function to penalize movement of nodes from their position in the previous layout. The necessary constraints for layout stability can be automatically determined from an existing layout by using the current positions of nodes and adding separation constraints to preserve the horizontal (left-right) and vertical (top-bottom) relationships in the network.

#### Radial layouts

**Figure 7:**
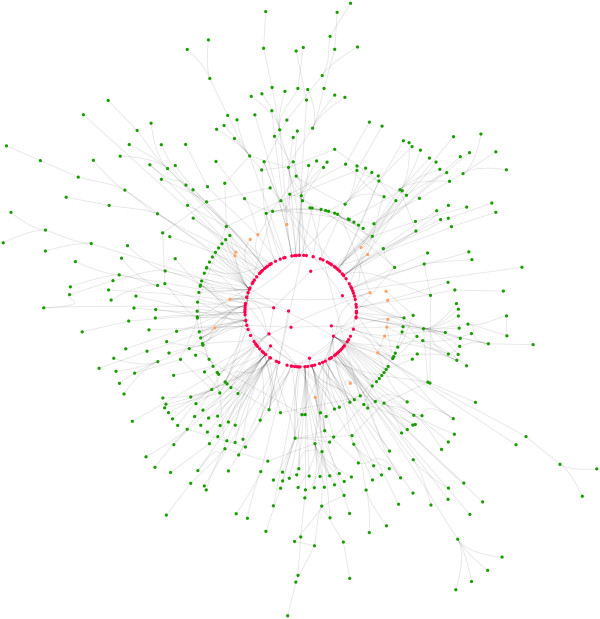
**Emphasizing relationships of interest: a protein interaction network with 3 radial band constraints (rings around the center) to place the most important proteins (red) at the center; see Figure 5(b) for the unconstrained version of the layout**. The importance of proteins can be given by different methods such as computationally by centrality analysis or experimentally by knock-out mutants.

Radial layouts can be used to emphasize the importance of nodes, which could be placed in the center of the diagram, see Figure 7. At first glance it seems that radial layouts cannot be generated with our algorithm. However, instead of constraints on Cartesian co-ordinates we can allow constraints over polar co-ordinates (or at least radii) and the layout algorithm works basically unchanged. In this case separation constraints separate nodes by distance from the origin. Ordering or ranking of nodes and therefore the separation constraints can be, for example, given by experimental data or computed with network centralities [36].

## Results and Discussion

### Network-specific layouts

Several types of biological networks exist such as gene regulatory, protein interaction and metabolic networks. These networks describe different aspects of biological processes and typically utilize quite different layouts so as to better highlight relevant information. Gene regulatory and signal transduction networks, for example, describe the cellular control of the protein synthesis during transcription as well as the communication within a cell to coordinate responses to external or internal changes. Typical visualization requirements are to show the temporal order of events and the representation of different cellular compartments (e. g. cytosol, mitochondrium, nucleus). Some methods for the visualization of these networks are described in [17,18]. Typical visualizations of protein-interaction networks emphasize the connectedness or clustering of proteins. Methods for their visualization are, for example, presented in [3,6]. The visualization of metabolic networks should typically emphasize the temporal order of reactions and distinguish several elements of a reaction: reactants (often divided into main- and co-reactants), products (main and co-products) and enzymes. Typical methods for the visualization of metabolic pathways are described in [4,7,10].

**Figure 8:**
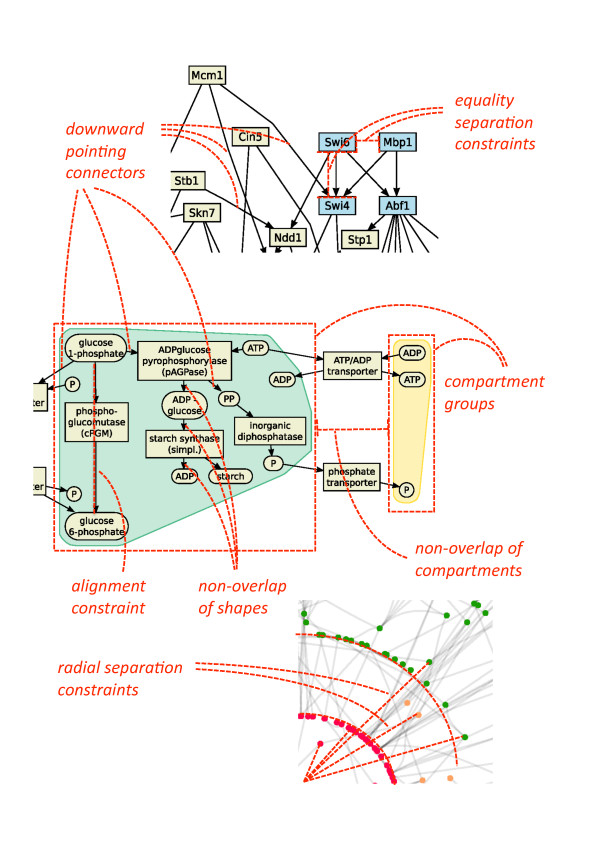
**Typical constraints used to produce the layouts in Figures 5-7**.

All these different drawing styles can be achieved with our algorithm. It provides a generic approach to network visualization, subsumes force-directed approaches [12,13], Sugiyama style layered layout [14], circular and grid drawings [9,16]. It has produced the drawings in Figure 5 and 6(b) which previously had to be produced using totally different algorithms. For example, in Figure 5(a) the visualization of a gene regulatory network which shows regulatory events from top to bottom is presented. The layout style is similar to layered layouts such as in the graphical interface of the TransPath database [37]. Figure 5(b) shows a protein interaction network in a force-directed like drawing style which is typical for graphical representations of such networks. It nicely presents the overall structure of the network and emphasizes highly connected or clustered proteins. Figure 6(b) shows a metabolic pathway in a mixture of Sugiyama style layered and grid layout. Typically, such pathway visualizations should emphasize the temporal order of reactions, distinguish several elements of a reaction and obey compartments. Again, the presented layout method supports these constraints and gives layouts similar to those obtained with established methods for the visualization of metabolic networks. Figure 8 shows typical constraints used to produce these layouts.

### Consideration of hierarchical information and network analysis results

The layout approach is not only able to visualize different biological networks in their typical style. It is more powerful than most existing layout algorithms and in particular able to handle convex and rectangular clusters allowing network hierarchy or additional information to be emphasized. As an example the diagram in Figure 6(b) is an automatic drawing of a metabolic pathway with compartments which previously had to be drawn by hand, such as in Figure 6(a).

The method also allows the user to customize the layout to emphasize relationships of interest, for example, to explore network analysis results such as network centrality analysis, network motif investigation and network clustering. For example, Figure 7 shows a protein interaction network arranged using the constrained stress majorization method with 3 radial band constraints such that the most important proteins (red) are at the center and less important ones (green) are at the outer border. The unconstrained version of the layout is shown in Figure 5(b). Additionally, Figure 5(a) shows a layered drawing of a gene regulatory network with two bi-fan motifs highlighted and automatically drawn similarly.

## Conclusion

We present a new method for producing high-quality visualizations of a wide range of biological networks with different layout requirements. Our approach is based on constrained graph layout and allows constraints to be used to capture drawing conventions found in biological literature as well as user-specified drawing requirements. In addition, most of the constraints can be automatically derived from the network structure, biological information or network analysis methods.

The generalization of different layout algorithms is desirable, but it comes at the price that a more general approach may lose the efficiency of specialized algorithms. A prototype implementation of our algorithm demonstrates that these methods are fast enough for use in interactive applications for networks with several hundred nodes and can lay out larger networks with a few thousand nodes in about one minute. The key to the efficiency of the proposed method is that projection on to separation constraints can be done efficiently using specialized algorithms [25]. We find that the main cost is in finding the optimal layout from the initial feasible layout and that the dominating cost in this is computing the quadratic approximation to the cost function since this is quadratic in the number of nodes.

This algorithm offers the ability to produce many of the fundamental popular drawing styles while allowing the flexibility of constraints to further tailor these layouts. In addition, the approach can handle multiple layout styles within a single drawing as well as mixed graphs (i. e. graphs with some directed edges). Constraints can also be used to provide stability to the layout and preserve the user's mental map when creating new layouts of existing networks. Finally, constraints offer the added benefit of producing drawings based on recognizable features of existing biological network visualizations such as those in the KEGG pathways database [38]. In this case, the KEGG layout as given by the KGML file could be used to derive orthogonal ordering constraints which will result in a layout of nodes similar to the relativ placement in the KEGG diagram.

Our paper focuses on molecular biological networks such as gene regulatory, protein interaction and metabolic networks. However, the presented method is very general and could also be adapted to other biological networks such as phylogenetic networks and food webs.

## Authors' contributions

TD, KM and FS designed the study; TD, KM and MW developed the constraint layout algorithm; TD and FS developed the constraint sets; TD and MW implemented the layout algorithm; FS evaluated the results. All authors wrote and approved the final manuscript.
